# Hesperidin as a Species-Specific Modifier of Aphid Behavior

**DOI:** 10.3390/ijms25094822

**Published:** 2024-04-28

**Authors:** Katarzyna Stec, Bożena Kordan, Jan Bocianowski, Beata Gabryś

**Affiliations:** 1 Department of Botany and Ecology, University of Zielona Góra, Szafrana 1, 65-516 Zielona Góra, Poland; 2 Department of Entomology, Phytopathology and Molecular Diagnostics, University of Warmia and Mazury in Olsztyn, Prawocheńskiego 17, 10-720 Olsztyn, Poland; bozena.kordan@uwm.edu.pl; 3 Department of Mathematical and Statistical Methods, Poznań University of Life Sciences, Wojska Polskiego 28, 60-637 Poznań, Poland; jan.bocianowski@up.poznan.pl

**Keywords:** pea aphid, bird cherry-oat aphid, green peach aphid, electrical penetration graph (EPG), antifeedants, biopesticides

## Abstract

Hesperidin is a highly bioactive natural flavonoid whose role in ecological interactions is poorly known. In particular, the effects of hesperidin on herbivores are rarely reported. Flavonoids have been considered as prospective biopesticides; therefore, the aim of the present study was to examine the influence of hesperidin on the host plant selection behavior of three aphid (Hemiptera: Aphididae) species: *Acyrthosiphon pisum* Harrris, *Rhopalosiphum padi* (L.), and *Myzus persicae* (Sulz.). The aphid host plants were treated with 0.1% and 0.5% ethanolic solutions of hesperidin. Aphid probing behavior in the no-choice experiment was monitored using electropenetrography and aphid settling on plants in the choice experiment was recorded. The results demonstrated that hesperidin can be applied as a pre-ingestive, ingestive, and post-ingestive deterrent against *A. pisum*, as an ingestive deterrent against *R. padi*, and as a post-ingestive deterrent against *M. persicae* using the relatively low 0.1% concentration. While in *A. pisum* the deterrent effects of hesperidin were manifested as early as during aphid probing in peripheral plant tissues, in *M. persicae,* the avoidance of plants was probably the consequence of consuming the hesperidin-containing phloem sap.

## 1. Introduction

**Figure 1:**
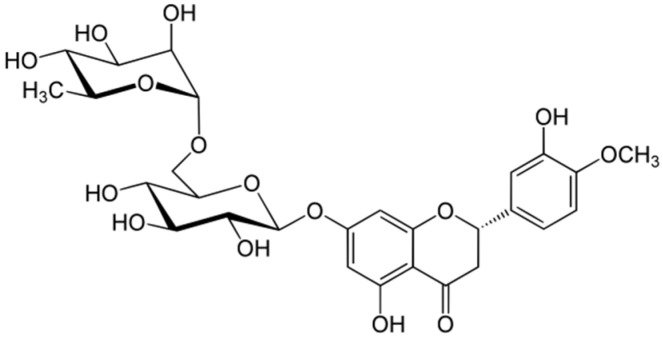
Chemical structure of hesperidin.

Hesperidin (3,5,7-trihydroxyflavanone 7-rhamnoglucoside) (Figure 1) belongs to the diverse group of flavonoids. In general, flavonoids occur in most plants and are involved in all kinds of ecological interactions, mainly in defenses against various abiotic and biotic stresses, but also as infochemicals in extraorganismal plant signaling [1,2,3,4,5]. Flavonoids are present in various tissues, cells, and sub-cellular compartments [3] and their biological functions in plants include the defense against UV-B radiation and pathogen infection, nodulation, and pollen fertility [6]. The roles of many flavonoids in plant–herbivore relationships have been well documented [1,7,8,9,10]. These plant metabolites can have negative effects on non-adapted herbivores, may reduce the nutritive value of the food, or may act as feeding deterrents or toxins [11,12,13]. The flavonoid mode of action on microorganisms and insects probably arises from an interference with important cellular processes and structures, yet this is not fully understood [14]. In view of these activities, flavonoids have been considered as prospective biopesticides [14,15,16].

Hesperidin occurs mainly in the flavedo, albedo, segment membranes and juice sacs of the fruits of plants of the genus *Citrus* (Rutaceae) [17,18], but it has also been recorded from Fabaceae, Betulaceae, and Lamiaceae [19,20,21]. Hesperidin has attracted a lot of attention as one of the most interesting and promising bioflavonoids for application in traditional medicines and as a combination product [20,21,22]. It is safe in application to humans and without side effects even during pregnancy [21]. Various pharmacological activities of hesperidin have been reported, including antioxidant, antibacterial, antimicrobial, antiviral, anti-inflammatory, and anticarcinogenic properties [20,22,23].

In contrast, the roles of hesperidin in ecological interactions and its effects on herbivores in particular have rarely been explored and reported. At the molecular level, the available reports provide information that hesperidin alleviates oxidative stress in *Drosophila melanogaster* Meigen (Diptera: Drosophilidae) caused by the groundwater pollutant trichloroethylene [24]. At the organismal level, the proven activities of hesperidin in plant–herbivore interactions include its roles as host plant recognition cue and oviposition stimulant for *Papilio protenor* Cramer and *P. xuthus* L. (Lepidoptera: Papilionidae) [1,7,25], defense chemical in *Citrus* x *sinensis* (L.) Osbeck against *Xylella fastidiosa* Wells et al. (Bacteria: Xanthomonadaceae) [26], *Phytophthora citrophthora* (R.E. Sm. and E.H. Sm.) Leonian (Oomycetes: Peronosporaceae) and *Candidatus liberibacter* Fagen et al. (Bacteria: Rhizobiaceae) [27,28], and in *C. aurantium* L. against *Aphis punicae* Passerini (Hemiptera: Aphididae) and *Planococcus citri* Risso (Hemiptera: Pseudococcidae) [29]. Hesperidin, applied as hesperidin-Mg complex, showed insecticidal activity against *Spodoptera frugiperda* (Lepidoptera: Noctuidae) and *Bemisia tabaci* (Hemiptera: Aleyrodidae) [30,31]. In the same study [30], the repellent activity of the hesperidin-Mg complex towards *Myzus persicae* (Hemiptera: Aphididae) was reported with the reservation that it required further evaluation. Hesperidin was also reported as a weak antifeedant when offered to aphids (Hemiptera: Aphididae) *M. persicae* and *Schizaphis graminum* in artificial diets [32]. The practical lack of knowledge on the effect of hesperidin on insect herbivores is surprising: the data are fragmentary and refer mainly to herbivores associated with citrus plants. Hesperidin is a rather unique flavonoid, the natural occurrence of which is limited to a narrow range of plant species. As such, it should be considered as a prospective herbivore-limiting factor, especially in relation to monophagous and oligophagous insects that do not use this flavonoid as the host plant recognition cue.

The aim of the present study was to investigate in detail the effect of hesperidin on host plant selection behavior of three species of aphids that vary in host plant specialization that determines their sensitivities to plant allelochemicals. The species studied were the oligophagous pea aphid *Acyrthosiphon pisum* Harris, which is the non-host alternating specialist on Fabaceae, the bird cherry-oat aphid *Rhopalosiphum padi* (L.), which is the host-alternating oligophagous specialist on Poaceae, and the highly polyphagous green peach aphid *Myzus persicae* (Sulz.). We hypothesized that hesperidin, absent in their preferred host plants, may alter the ability of aphids to recognize and accept these plants as hosts. We concentrated on behavioral aspects of aphid host plant selection process, specifically on aphid probing and settling behaviors. Our studies were carried out under semi-natural conditions in no-choice and choice situations. In the no-choice experiment, tethered aphids were offered their preferred host plants untreated and treated with hesperidin. Then, the aphid stylets’ movements in plant tissues were monitored using the technique of electropenetrography (Electrical Penetration Graph, EPG). This experiment was to reveal whether hesperidin had any deterrent influence on individual phases of probing in specific plant tissues. In the choice experiment, the freely moving aphids could choose between hesperidin-treated and untreated plant leaves. This experiment was designed to establish the potency and durability of hesperidin antifeedant activity.

## 2. Results

### 2.1. Acyrthosiphon Pisum

#### 2.1.1. Aphid Probing Behavior (EPG No-Choice Experiment)

**Figure 2:**
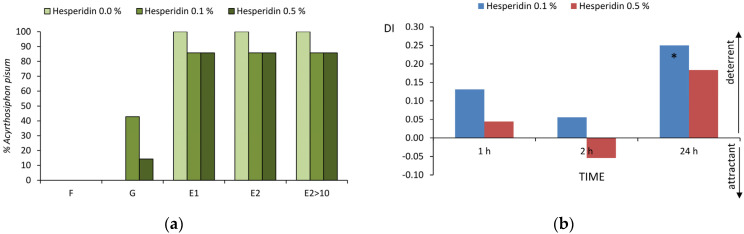
Behavior of *Acyrthosiphon pisum* on *Pisum sativum* treated with ethanolic solutions of hesperidin. (**a**) Frequency of non-pathway probing activities expressed as the percentage of aphids which showed the specific activity (EPG; no-choice experiment); (**b**) effect of hesperidin on aphid settling expressed as deterrence indices (DI; choice experiment) after 1, 2, and 24 h; asterisks indicate significant differences in aphid settling on control vs. hesperidin-treated leaves (*p* < 0.05; Student *t*-test).

The electropenetrography of *Acyrthosiphon pisum* on *Pisum sativum* revealed waveforms that visualized three major phases in aphid probing activities irrespective of treatment: no probing (aphid stylets outside the plant), probing in non-vascular tissues epidermis and mesophyll, and probing in vascular tissues xylem and phloem. However, several significant differences in probing behavior were revealed, depending on plant treatment with 0.1% and/or 0.5% ethanolic solutions of hesperidin (Table 1, Table S1, Table 2 and Table S2; Figure 2a).

The pea aphid probing on hesperidin-treated plants differed from the pea aphid probing on control plants in the frequency of occurrence and duration of specific activities. All aphids in all treatments showed pathway activities ‘C’. No xylem sap ingestion activity ‘G’ occurred on control plants, while on 0.1% and 0.5% hesperidin-treated plants, 43% and 14% of aphids ingested sap from xylem vessels and the average duration of ‘G’ was 1 h and 2 h, respectively. All aphids on control plants showed phloem phase, including the sustained sap ingestion, while on hesperidin-treated plants, 14% of aphids failed to locate sieve elements, regardless of the applied hesperidin concentration. On 0.1% and 0.5% hesperidin-treated plants, the total durations of phloem phase were 1.7 and 2.1 times shorter, and the total durations of phloem sap ingestion were 1.8 and 2.2 times shorter than on control, respectively. The individual bouts of sap ingestion were 2.8 times shorter on both 0.1% and 0.5% hesperidin-treated plants than on control. At the same time, the contribution of salivation to the phloem phase was 1.5 and 2.6 higher on 0.1% and 0.5% hesperidin-treated plants as compared to control, respectively (Table 1 and Table S1; Figure 2a).

Aphid probing on hesperidin-treated plants also differed significantly in the timing of individual activities in reference to control. The periods to the first phloem phase and the first phloem sap ingestion phase from start of EPG and from the first probe were 2.8 and 2.2 times longer on 0.1% and 0.5% hesperidin-treated plants, respectively. The duration of pathway phase preceding the first phloem phase in that probe was similar in all aphids, but the first sap ingestion phase and the first sustained sap ingestion phase in these probes were delayed by 10–12 min on 0.5% hesperidin-treated plants than on control and 0.1% hesperidin-treated plants. The first phloem phases were 2.0 and 3.7 times shorter on 0.1% and 0.5% hesperidin-treated plants, respectively. The potential E2 index was 1.5 and 2.1 higher on control plants than on 0.1% and 0.5% hesperidin-treated plants, respectively (Table 2 and Table S2).

#### 2.1.2. Aphid Settling (Choice Experiment)

Significantly fewer pea aphids settled on pea leaves treated with 0.1% ethanolic solution of hesperidin 24 h after hesperidin application; the value of deterrence index (DI) was 0.25. No significant differences in aphid settling occurred after the application of 0.5% ethanolic solution of hesperidin, but a trend in aphid preference for untreated leaves was observed 24 h after application (DI = 0.18) (Figure 2b).

### 2.2. Rhopalosiphum Padi

#### 2.2.1. Aphid Probing Behavior (EPG No-Choice Experiment)

**Figure 3:**
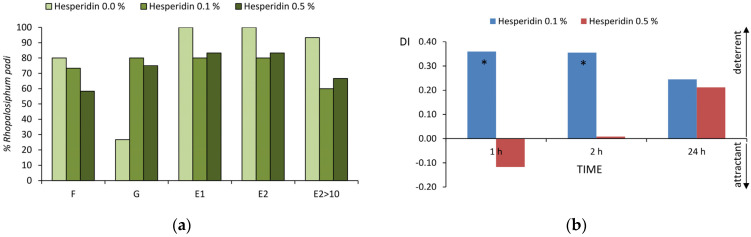
Behavior of *Rhopalosiphum padi* on *Avena sativa* treated with ethanolic solutions of hesperidin. (**a**) Frequency of non-pathway probing activities expressed as the percentage of aphids which showed the specific activity (EPG; no-choice test); (**b**) effect of hesperidin on aphid settling expressed as deterrence indices (DI; choice test) after 1, 2, and 24 h; asterisks indicate significant differences in aphid settling on control vs. hesperidin-treated leaves (*p* < 0.05; Student *t*-test).

The EPG waveforms recorded for *Rhopalosiphum padi* on *Avena sativa* represented no probing, probing activities in non-vascular tissues epidermis and mesophyll, and probing activities in vascular tissues xylem and phloem. Depending on plant treatment with 0.1% and/or 0.5% ethanolic solutions of hesperidin, significant differences in frequency of occurrence and duration of aphid stylet activities were recorded (Table 3, Table S3, Table 4 and Table S4; Figure 3a).

The total duration of no probing was 2.6 and 1.4 times longer on 0.1% and 0.5% hesperidin-treated plants than on control (Table 3 and Table S3), respectively. Probing activities were divided into pathway, derailed stylet activities, xylem sap ingestion, phloem salivation, and phloem sap ingestion, which occurred with different frequencies and durations, depending on the treatment. All *R. padi* in all treatments showed pathway activities ‘C’, and no significant differences between either treatment and control were observed in total duration and duration of individual bouts of ‘C’ (Table 3 and Table S3). The frequencies of the remaining non-phloem activities ‘F’ and ‘G’ were variable, depending on the treatment. On control plants, 80% *R. padi* showed activity ‘F’ and 27% showed activity ‘G’. On 0.1% and 0.5% hesperidin-treated plants, these frequencies were, respectively, 80% and 58% for ‘F’ and 80% and 75% for ‘G’ (Figure 3a). The durations of ‘F’ on hesperidin-treated plants were similar to control. In contrast, the total duration of ‘G’ was 3.3 times longer on 0.1% hesperidin-treated plants and 6.2 times longer on 0.5% hesperidin-treated plants. The individual bouts of xylem sap ingestion were of similar durations in all treatments (Table 3 and Table S3). The phloem phase occurred with various frequencies: in 80% and 83% aphids on 0.1% and 0.5% hesperidin-treated plants, respectively, and in all aphids on control (Figure 3a). The total durations of phloem phase and sap ingestion phase were 2.2 and 1.8 times longer on control plants than on 0.1% and 0.5% hesperidin-treated plants, respectively. The input of salivation to the phloem phase was similar in all aphids in all treatments (Table 3 and Table S3; Figure 3a).

There was a trend towards delay in reaching phloem vessels on hesperidin-treated plants: on control plants, aphids reached the first phloem phase in 1.9 h on average, while it was in 2.9 and 2.8 h on 0.1% and 0.5% hesperidin-treated plants, respectively (Table 4 and Table S4). The duration of pathway and other non-phloem activities within the probe which led to the first bout of sustained sap ingestion was shortest on control (21.7 min) and longest on 0.5% hesperidin-treated plants (56 min) (Table 4 and Table S4). The potential E2 index was 2.4 and 1.6 times higher on control plants than on 0.1% and 0.5% hesperidin-treated plants, respectively (Table 4 and Table S4).

#### 2.2.2. Aphid Settling (Choice Experiment)

Significant differences in the bird cherry-oat aphid preferences to settle on untreated oat leaves were recorded when aphids had a choice between untreated and 0.1% hesperidin-treated leaves. Aphids demonstrated their preferences 1 h and 2 h after having access to the treatment. The indices of deterrence were 0.36 at both the 1st and 2nd hour of observation (Figure 3b). No significant differences in aphid settling occurred after the application of 0.5% ethanolic solution of hesperidin. However, 1 h after application, 0.5% hesperidin showed a weak attractant quality, but 24 h after exposure, aphids tended to avoid the 0.5% hesperidin-treated leaves (Figure 3b).

### 2.3. Myzus Persicae

#### 2.3.1. Aphid Probing Behavior (EPG No-Choice Experiment)

**Figure 4:**
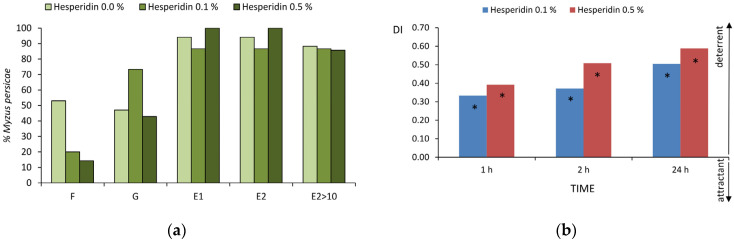
Behavior of *Myzus persicae* on *Brassica rapa* subsp. *pekinensis* treated with ethanolic solutions of hesperidin. (**a**) Frequency of non-pathway probing activities expressed as the percentage of aphids which showed the specific activity (EPG; no-choice); (**b**) effect of hesperidin on aphid settling expressed as deterrence indices (DI; choice) after 1, 2, and 24 h; asterisks indicate significant differences in aphid settling on control vs. hesperidin-treated leaves (*p* < 0.05; Student *t*-test).

Probing activities of the green peach aphid on untreated and hesperidin-treated cabbage comprised stylet penetration in non-phloem tissues and in the phloem. The duration of no probing was similar in all aphids. Pathway activities ‘C’ occurred in all aphids in all treatments, and the frequency and duration of this activity was similar in all individuals. Derailed stylet activities ‘F’ occurred in 53% of aphids on control plants, while on 0.1% and 0.5% hesperidin-treated plants, ‘F’ occurred in 21% and 14% aphids, respectively (Figure 4a). The number and total and mean durations of ‘F’ differed significantly among treatments: on 0.1% hesperidin-treated plants, the number and total duration of ‘F’ were 2.9 and 3.3 times lower than on control, respectively, and on 0.5% hesperidin-treated plants, the number and total and mean durations of ‘F’ were 5.4, 18.8, and 3.9 times lower than on control (Table 5 and Table S5). Xylem phase ‘G’ occurred in 47% of aphids on control plants, while on 0.1% and 0.5% hesperidin-treated plants, the frequency of ‘G’ was 73% and 43%, respectively (Figure 4a). The mean duration of ‘G’ was 1.9 and 1.7 times shorter on 0.1% and 0.5% hesperidin-treated plants than on control (Table 5 and Table S5). Phloem phase, sap ingestion phase, and sustained sap ingestion phase occurred in 94%, 87%, and 93% of aphids on control and on 0.1% and 0.5% hesperidin-treated plants (Figure 4a), respectively. Significant differences occurred in total and mean duration of phloem salivation preceding the first bout of sustained sap ingestion; the highest values occurred on 0.1% hesperidin-treated plants (Table 5 and Table S5).

The sequence of events in aphid probing was similar in all aphids in all treatments from the start until the end of the EPG (Table 6 and Table S6).

#### 2.3.2. Aphid Settling (Choice Experiment)

Freely moving aphids settled mainly on control untreated cabbage leaves. The deterrence indices for 0.1% hesperidin recorded after 1 h, 2 h, and 24 h were, respectively, 0.3, 0.4, and 0.5. The deterrence indices for 0.5% hesperidin recorded after 1 h, 2 h, and 24 h were, respectively, 0.4, 0.5, and 0.6 (Figure 4b).

## 3. Discussion

Secondary plant compounds can adversely affect three major phases in insect activities associated with feeding: the pre-ingestive, ingestive, and post-ingestive phases [33]. The impact of plant allelochemicals during the pre-ingestive phase is associated with host finding and host selection processes and involves gustatory receptors, while during the ingestive phase, the effects are related to the transport of food as well as the release and digestion by salivary enzymes. The post-ingestive effects are usually delayed in time and refer to various aspects of digestion and absorption of food. The presented classification of deterrent effects of allelochemicals was originally established for insect herbivores that consume plant material using their chewing mouthparts equipped with chemosensillae [33]. Aphids are plant sap-consuming herbivores with specialized sucking-piercing mouthparts that lack external contact chemoreceptors; the gustatory organ is located in the hypopharynx [34]. Henceforth, the pre-ingestive and ingestive phases of host plant selection by aphids require the insertion of the mouthparts’ stylets into plant tissues. Consequently, this activity and the possible immediate responses of aphids to plant allelochemicals are hidden from the human eye. Electropenetrography is the technique which allows an insight into pre-ingestive and ingestive aphid behaviors [35,36]. The parameters describing aphid behavior during probing such as the total time of probing, the duration and frequency of phloem sap ingestion events, the number of probes, etc., are good indicators of plant suitability or the interference in probing by chemical or physical factors present in individual plant tissues or administered exogenously on plant surface [37]. The post-ingestive effects of plant allelochemicals on aphid foraging behavior can be determined by monitoring aphid probing and preferences of the free-moving aphids in settling on plants [38].

The present study showed that the pre-probing behavior was similar in all aphids: all aphids initiated probing shortly after gaining access to experimental plants. This finding is consistent with previous reports on aphid predisposition to probe in any substrate, provided that no deterrent constituents are present on the surface [39]. Significant alterations in the foraging behavior occurred after the aphids inserted stylets into plant leaves treated with 0.1% and/or 0.5% ethanolic solutions of hesperidin.

The modification of aphid behavior during the pre-ingestive phase concerned different aspects of probing in non-vascular tissues epidermis and mesophyll. In the case of *A. pisum*, the time to cross the barrier of epidermis and mesophyll to reach phloem vessels and start sustained feeding was significantly longer on hesperidin-treated *P. sativum*. This was caused by the discontinuation of the relatively long probes, i.e., probes including pathway longer than 3 min, which means that aphids reached beyond epidermis before the stylets were withdrawn [40]. At the same time, the pea aphids spent more time on no probing activities on hesperidin-treated plants than on control. Nevertheless, the probes were repeated and finally, most of the aphids reached phloem on all plants. The successful probes—those that ended in the sieve elements—included the pathway of similar duration on treated and untreated plants. No individual of *A. pisum* showed activity ‘F’ on any plant, which reflects the lack of difficulties in mechanical work of the stylets in the apoplast [35]. No effects of hesperidin concentration on the pea aphid probing in non-vascular tissues were observed. In *R. padi,* statistical analysis did not detect significant differences in the time to reach sieve elements from the onset of probing, but a trend towards an increase in the duration of the pre-phloem period occurred in aphids on hesperidin-treated *A. sativa*. Nevertheless, the proportion of aphids that reached sieve elements was reduced on hesperidin-treated plants as compared to control. Evident differences were recorded when analyzing the duration of the pathway that directly preceded the first contact with sieve elements within a probe: it was twice as long on 0.1% hesperidin-treated plants, but similar on 0.5% hesperidin-treated plants in respect to control. The incidence of activity ‘F’ in apoplast was the highest on control and the lowest on 0.5% hesperidin-treated plants. In *M. persicae*, no differences in the probing behavior occurred during the pre-phloem phase, except the frequency of activity ‘F’, which was the highest on control and the lowest on 0.5% hesperidin-treated plants. The pathway activity comprises extracellular movements of stylets and brief punctures of cells adjacent to stylet route for gustatory purposes [35,41]. It may be stated that the more frequent events of termination of pathway probes on hesperidin-treated plants in relation to control were caused by the deterrent properties of this flavonoid. Hesperidin might have been detected in the sap samples acquired by aphids during the pathway cell punctures, as it has been found in the cases of various exogenously applied allelochemicals [36,37,38].

The ingestive phase in aphid probing embraces the uptake of sap from phloem and/or xylem vessels [35]. The uptake of the phloem sap is always preceded by a shorter or longer bout of watery saliva secretion into the sieve element [42]. The role of the watery saliva is to prepare and adjust the sieve element for aphid feeding by blocking or eliminating plant defense mechanisms [43]. The duration of salivation and the contribution of this activity to the phloem phase reflects the potency of plant defense factors located in the phloem [42,43,44]. In *A. pisum*, the contribution of salivation to the phloem phase was the lowest on control *P. sativum* and the highest on 0.5% hesperidin-treated plants, while in *R. padi* and *M. persicae,* no differences among treatments occurred. In *A. pisum* and *R. padi*, the total and mean durations and the contribution of sap ingestion activity to all probing activities were twice as low on hesperidin-treated plants than on control, and no effect of hesperidin concentration was recorded. In *M. persicae*, no significant differences related to sap ingestion activities occurred among treatments.

While the ingestion of the nutrient-rich phloem sap is actually the feeding activity and reflects plant acceptance, the ingestion of the xylem sap that contains mostly water occurs usually under stress and probably reflects the inability to use phloem resources due to the presence of negative factors in the plant at the stage before the aphids reach the phloem [44]. In *A. pisum*, *R. padi,* and *M. persicae,* the xylem sap ingestion activity was the most frequent in aphids on 0.1% hesperidin-treated plants. However, the duration of individual bouts and the contribution to the total probing activities differed among aphid species, and in some cases, these variables were hesperidin concentration-dependent. In *A. pisum*, the duration of individual bouts of xylem phase was extended in aphids on 0.1% and on 0.5% hesperidin-treated plants, while in *R. padi* and *M. persicae*, the bouts of xylem sap ingestion were longer on hesperidin-treated plants, but no effect of hesperidin concentration was observed.

The behavior of aphids during the post-ingestive phase reflects the level of suitability of a plant for feeding, settling, and reproduction [33]. On suitable hosts, aphids may continue phloem sap ingestion for many hours without interruption, while on less accepted plants or on non-hosts, the bouts of phloem sap ingestion are relatively short and interrupted by periods of pathway activities within the same probe, or even by periods of no probing which follow the withdrawal of the stylets [45]. The EPG experiment in the present study showed that the proportion of time spent on phloem sap ingestion after the beginning of the first bout of sustained sap ingestion (the ‘potential E2 index’) changed depending on the treatment with hesperidin and was aphid species-dependent. In *A. pisum* and *R. padi*, the values of pE2 index were reduced on hesperidin-treated plants, while in *M. persicae,* it was not the case. In *A. pisum* and *R. padi*, no hesperidin concentration-effect was observed. The free-choice experiment showed noticeable differences in response to hesperidin treatments among aphid species. Significant differences in preference to settle on untreated plants were recorded 24 h after treatment with 0.1% hesperidin in *A. pisum* and 1 and 2 h in *R. padi*. In *M. persicae*, the deterrent effects of both 0.1% and 0.5% hesperidin were recorded 1 h, 2 h, and 24 h after aphids gained access to plants. The avoidance of the treated leaves during settling might have been the delayed effect of consuming the toxic sap from hesperidin-treated leaves, as the ingestion of the phloem sap was not obstructed. This explanation, though, needs further study.

In summary, the results of the present study indicate that hesperidin can be ascribed to all three functional groups of feeding deterrents, respectively, the pre-ingestive, ingestive, and the post-ingestive groups, depending on aphid species and the applied concentration. Hesperidin can be applied as a pre-ingestive, ingestive, and post-ingestive deterrent against *A. pisum*, as an ingestive deterrent against *R. padi*, and as a post-ingestive deterrent against *M. persicae*. In all cases, hesperidin can be applied at a relatively low 0.1% concentration, as an increase in the amount of hesperidin did not evoke significantly stronger effects on aphid probing behavior as compared to 0.1% concentration. The results of the present study also demonstrate that the oligophagous *A. pisum* was the most sensitive to the application of hesperidin, and the polyphagous *M. persicae* was the least sensitive. While in *A. pisum* the deterrent effects of hesperidin were manifested as early as during aphid probing in peripheral plant tissues, in *M. persicae*, the avoidance of plants was probably the consequence of consuming the hesperidin-containing phloem sap.

## 4. Materials and Methods

### 4.1. Cultures of Plants and Aphids

Laboratory clones of *Acyrthosiphon pisum*, *Myzus persicae*, and *Rhopalosiphum padi* were maintained on *Pisum sativum* cv. Milwa (Hodowla Roślin Smolice Sp. z o.o. Grupa IHAR, Smolice 146, 63-740 Kobylin, Poland), *Brassica rapa* ssp. *pekinensis* cv. Hilton (World of Flowers Sp. z o.o., ul. Sulejkowska 56/58, 215, 04-157 Warszawa, Poland), and *Avena sativa* cv. Komfort (Hodowla Roślin Strzelce Sp. z o.o. Grupa IHAR, ul. Główna 20, 99-307 Strzelce, Poland), respectively, in the laboratory at 20 °C, 65% r.h., and L16:D8 photoperiod. Aphid clones have been maintained in the laboratory of Department of Botany and Ecology, University of Zielona Góra, Poland for at least 10 years. One- to seven-day old apterous aphid females and three-week-old plants were used for the experiments. Plants used for experiments were the same plant species and cultivars that were used for the rearing of aphids. All experiments were carried out under the same conditions of temperature, relative humidity, and photoperiod. The bioassays were started at 10–11 a.m.

### 4.2. Application of Hesperidin

Hesperidin (≥80% HPLC) was purchased from Sigma–Aldrich (Poznań, Poland). The flavonoid was dissolved in 70% ethanol to obtain 0.1% and 0.5% solutions. For the aphid probing behavior experiment (no-choice test), hesperidin was applied on the adaxial and abaxial leaf surfaces by immersing one leaf of an intact plant in the ethanolic solution of a given concentration for 30 s. Control leaves of similar size on the control intact plants were immersed in 70% ethanol that was used as a solvent for the studied compound. For the aphid settling success experiment (choice-test), hesperidin was applied on the adaxial and abaxial leaf surfaces by immersing the cut leaves in the ethanolic solution of a given concentration for 30 s. Control leaves of similar size were immersed in 70% ethanol.

All experiments were performed 1 h after the compound application to allow for the evaporation of the solvent.

### 4.3. Aphid Probing Behavior (No-Choice Experiment)

Aphid probing (aphid stylet penetration in plant tissues) was monitored using the electronic penetration graph technique (electropenetrography) known as EPG, which is frequently employed in insect–plant relationship studies considering insects with sucking-piercing mouthparts. In this experimental setup, aphids and plants are parts of an electric circuit, which is completed when the aphid inserts its stylets into the plant. Weak voltage is supplied in the circuit, and all changing electric properties are recorded as EPG waveforms that can be correlated with aphid activities and stylet position in plant tissues. In the present study, aphids were attached to a golden wire electrode with conductive silver paint and starved for 1 h prior to the experiment. Probing behavior of 20 apterous females per studied flavonoid concentration/aphid combination was monitored for 8 h continuously with four-channel DC EPG recording equipment. Each aphid was given access to a freshly prepared plant leaf of an intact plant. Each plant–aphid set was considered as a replication and was tested only once. The number of replications (EPG recordings) for each plant treatment was 24. Recordings that terminated due to aphid falling from the plant or where EPG signal was unclear were discarded from analysis. Only the replications that included complete 8 h recordings were kept for analysis. All experiments were carried out under the same conditions of temperature, relative humidity (r.h.), and photoperiod as those used for the rearing of plants and aphids. All bioassays started at 10:00–11:00 h MEST (Middle European Summer Time).

Signals were saved on the computer and analyzed using the PROBE 3.1 software provided by W.F. Tjallingii (www.epgsystems.eu, accessed on 20 August 2022; Wageningen 6703 CJ, The Netherlands). The following aphid behaviors were distinguished: no penetration (waveform ‘np’—aphid stylets outside the plant), pathway phase-penetration of non-phloem tissues (waveforms ‘ABC’), derailed stylet movements (waveform ‘F’), salivation into sieve elements (waveform ‘E1’), ingestion of phloem sap (waveform ‘E2’), and ingestion of xylem sap (waveform ‘G’). The E1/E2 transition pattern was split in two between E1 and E2. The waveform patterns that were not terminated before the end of the experimental period (8 h) were included in the calculations. All variables were processed using the EPG Excel Data Workbook produced by Sarria et al. [46]. The parameters derived from EPGs were analyzed according to their frequency and duration in a configuration related to activities in peripheral and vascular tissues. In non-sequential parameters, when a given waveform had not been recorded for an individual, the duration of that waveform was given the value of 0. In sequential parameters, when parameters related to phloem phase (E1 or E2) were involved, only aphids that reached phloem phase were included in the statistical analysis.

### 4.4. Aphid Settling Success (Choice-Experiment)

Aphids settle on a plant only when they accept it as a food source [47]. Therefore, the number of aphids that settle and feed on a given substrate is a good indicator of its suitability. This bioassay allows studying aphid host preferences under semi-natural conditions. Aphids are given free choice between control and treated leaves. In the present study, aphids were placed in the Petri dish along the line that divided the arena into two halves so that aphids could choose between treated (on one half of a Petri dish) and control leaves (on the other half of the dish). Aphids that settled, i.e., they did not move and the position of their antennae indicated feeding [48] on each leaf were counted at 1 h, 2 h, and 24 h intervals after access to the leaves (8 replicates, 20 viviparous apterous females/replicate). Aphids that did not settle on any of the leaves were discarded from calculations.

### 4.5. Statistical Analysis

EPG parameters describing aphid probing behavior (no-choice test) were calculated manually and individually for every aphid, and the mean and standard errors were subsequently calculated using the EPG analysis Excel worksheet created for this study. The results were statistically analyzed using ANOVA (Statistica 13.3 package) [13]. Fisher’s least significant differences (LSDs) were estimated at the 0.05 significance level to identify significant differences between individual traits. Homogeneous groups were designated based on these LSD values. The data deriving from the choice-test for freely moving aphids (aphid settling deterrent activity) were analyzed using Student’s *t*-test. If aphids showed clear preference for the leaf treated with the tested compound (*p* < 0.05), the compound was described as having attractant properties. If aphids settled mainly on the control leaf (*p* < 0.05), the compound tested in the respective choice-test was stated a deterrent. The relative index of deterrence (DI) was calculated according to the formula DI = (C − T)/(C + T), where C is the number of aphids that remained on control leaf, and T is the number of aphids that remained on the treated leaf. The value of DI ranged between “+1” (ideal deterrent) and “−1” (ideal attractant) [38].

## Figures and Tables

**Table 1:** Probing behavior of *Acyrthosiphon pisum* on *Pisum sativum* treated with ethanolic solutions of hesperidin in the EPG no-choice test: non-sequential EPG parameters.

EPG Variable	Hesperidin 0.0%		Hesperidin 0.1%		Hesperidin 0.5%		LSD_0.05_	*p*-ANOVA
	n ^1^	Mean ± SEM	n ^1^	Mean ± SEM	n ^1^	Mean ± SEM		
No probing								
Total duration of np	22	1354 a ± 222.2	14	2976 a ± 759.3	14	3466 a ± 1288.2	2299.2	0.098
Number of np	22	15.5 a ± 1.9	14	20.3 a ± 4.0	14	19.7 a ± 3.4	9.06	0.409
Mean duration of np	22	80.5 a ± 7.4	14	195.5 a ± 71.9	14	238.4 a ± 129.3	222.1	0.252
Probing								
Total probing time	22	27,446 a ± 222.2	14	25,824 a ± 759.3	14	25,334 a ± 1288.2	2299.2	0.098
Number of probes	22	15.5 a ± 1.9	14	20.1 a ± 4.0	14	19.7 a ± 3.5	9.12	0.442
Number of short probes (C < 3 min)	22	8.0 a ± 1.4	14	11.3 a ± 3.0	14	9.8 a ± 2.4	6.69	0.542
Pathway phase								
Total duration of C	22	10,443 a ± 918	14	14,009 a ± 1681	14	14,205 a ± 1523	4038.4	0.063
Number of C	22	19.7 a ± 2.1	14	23.1 a ± 4.1	14	23.1 a ± 3.5	9.49	0.632
Mean duration of C	22	608.4 a ± 52.9	14	795.5 a ± 152.4	14	708.3 a ± 81.1	287.5	0.35
Proportion of probing spent in C (%)	22	38.4 b ± 3.5	14	54.5 a ± 6.5	14	54.5 a ± 6.5	15.33	0.014
Derailed stylet activities								
Total duration of F	22	0 a ± 0	14	0 a ± 0	14	0 a ± 0	*	-
Number of F	22	0 a ± 0	14	0 a ± 0	14	0 a ± 0	*	-
Mean duration of F ^2^	22	0 a ± 0	14	0 a ± 0	14	0 a ± 0	*	-
Proportion of probing spent in F (%)	22	0 a ± 0	14	0 a ± 0	14	0 a ± 0	*	-
Xylem phase								
Total duration of G	22	0 a ± 0	14	2005 a ± 918.3	14	2949 a ± 2028.1	3331.2	0.13
Number of G	22	0 a ± 0	14	0.5 a ± 0.2	14	0.5 a ± 0.4	0.618	0.108
Mean duration of G ^2^	22	0 c ± 0	6	3654 b ± 735	2	7677 a ± 4151	2687.5	0.011
Proportion of probing spent in G (%)	22	0 a ± 0	14	9.1 a ± 4.5	14	10.6 a ± 7.3	13.14	0.143
Phloem phase: general								
Total duration of phloem phase E (E1 + E2)	22	17,003 a ± 1047	14	9810 b ± 1922	14	8180 b ± 1555	4461.8	<0.001
Total duration of E1	22	527.9 a ± 106	14	437.9 a ± 125.5	14	572.3 a ± 160.4	395.9	0.784
Total duration of E2	22	16,476 a ± 1084	14	9372 b ± 1881	14	7608 b ± 1531	4455.3	<0.001
Phloem phase: salivation (E1)								
Number of E1	22	8 a ± 1.3	14	6.9 a ± 1.9	14	6.2 a ± 1.2	4.561	0.673
Mean duration of E1 ^2^	22	69.1 a ± 11.2	12	59.3 a ± 15.9	12	83.6 a ± 13.9	41.1	0.493
Number of single E1 ^2^	22	0.2 a ± 0.1	12	0.1 a ± 0.1	12	0.3 a ± 0.1	0.3583	0.723
Total duration of E1 followed by E2 ^2^	22	383.3 a ± 63.7	12	338.8 a ± 62	12	432.7 a ± 78.4	207.7	0.663
Total duration of E1 followed by E2 > 10 min ^2^	22	211.9 a ± 28.7	12	251.7 a ± 47.2	12	254.8 a ± 51.3	117.6	0.644
Duration of the E1 followed by 1st E2 ^2^	22	62.8 a ± 17.5	12	98 a ± 27.4	12	96 a ± 20.4	63.2	0.357
Duration of the E1 followed by 1st E2 > 10 min ^2^	22	61.4 a ± 17.3	12	96.8 a ± 27.5	12	121.8 a ± 26.2	66.7	0.131
Contribution of E1 to phloem phase (%) ^2^	22	3.6 b ± 0.7	12	5.2 ab ± 1.4	12	9.6 a ± 2.5	4.222	0.015
Proportion of probing spent in E1 (%)	22	1.9 a ± 0.4	14	1.6 a ± 0.5	14	2.4 a ± 0.6	1.475	0.58
Phloem phase: sap ingestion (E2)								
Number of E2	22	6.9 a ± 1.0	14	6 a ± 1.7	14	4.3 a ± 0.8	3.753	0.327
Number of E2 > 10 min	22	4 a ± 0.4	14	3.6 a ± 0.6	14	2.9 a ± 0.7	1.729	0.345
Mean duration of E2 ^2^	22	5153 a ± 1393	12	1826 a ± 474.8	12	1964 a ± 570.7	3526.5	0.057
Duration of the longest E2 ^2^	22	9178 a ± 1337.4	12	4342 b ± 848.8	12	4277 b ± 810.8	3690.8	0.004
Proportion of probing spent in E2 (%)	22	59.7 a ± 3.7	14	34.8 b ± 6.8	14	29.9 b ± 5.4	15.64	<0.001

^1^ Number of replications; ^2^ only the EPG recordings that included a particular waveform were included in calculations; np—no probing (aphid stylets outside the plant tissues); C—pathway activity (extracellular stylet penetration with potential drops, i.e., short cell punctures); F—derailed stylet activities (difficulties in penetration); G—xylem phase (ingestion of xylem sap); E—phloem phase including E1 (phloem salivation) and E2 (phloem sap ingestion); E2 > 10 min—sustained ingestion of phloem sap. Time and duration of various stylet activities are given in seconds. LSD_0.05_—least significant difference at the 0.05 significance level. * *p* < 0.05. Different letters in rows show significant differences at *p* < 0.05 (ANOVA). Shading indicates variables that are significantly different between treatments.

**Table 2:** Probing behavior of *Acyrthosiphon pisum* on *Pisum sativum* treated with ethanolic solutions of hesperidin: sequential EPG parameters.

EPG Variable	Hesperidin 0.0%		Hesperidin 0.1%		Hesperidin 0.5%		LSD_0.05_	*p*-ANOVA
	n ^1^	Mean ± SEM	n ^1^	Mean ± SEM	n ^1^	Mean ± SEM		
Start of EPG								
Time to 1st probe from start of EPG	22	81.7 a ± 22.8	14	40.1 a ± 14	14	131.6 a ± 87.3	143	0.442
Duration of 1st probe	22	1913 a ± 761.4	14	333 a ± 152.5	14	1382 a ± 605.8	2041.7	0.237
Duration of the second nonprobe period	22	48.2 a ± 7.9	14	139.6 a ± 101.4	14	71.3 a ± 16.7	154.9	0.422
Duration of 2nd probe	22	1436 a ± 694.9	14	977 a ± 638.6	14	2354 a ± 1439.7	2880.6	0.619
Before 1st phloem phase								
Time from start of EPG to 1st E ^2^	22	3900 b ± 638	14	10,741 a ± 2487	14	8653 ab ± 2460	5450.6	0.019
Time from 1st probe to 1st E ^3^	22	3819 b ± 638	14	10,700 a ± 2478	14	8521 ab ± 2407	5388.3	0.017
Time from the beginning of that probe to 1st E ^4^	22	1248 a ± 81.7	12	1461 a ± 160.5	12	1489 a ± 311	510	0.494
Number of probes to the 1st E1	22	6 a ± 1	12	10.4 a ± 2.1	12	8.2 a ± 2.5	5.034	0.151
Duration of nonprobe period before the 1st E	22	411 b ± 123.7	14	1743 a ± 722	14	898 ab ± 314.6	1214.7	0.061
Duration of the shortest C wave before E1 ^4^	22	986 a ± 75.7	12	1274 a ± 137.5	12	1217 a ± 142.5	325.2	0.107
1st phloem phase								
Duration of 1st phloem phase E ^4^	22	5284 a ± 1416	12	2639 ab ± 768.3	12	1438 b ± 337.9	3711.9	0.063
Before 1st sap ingestion phase E2								
Time from start of EPG to 1st E2 ^5^	22	4180 b ± 654	14	10,835 a ± 2488	14	10,709 a ± 2613	5619.6	0.012
Time from 1st probe to 1st E2 ^6^	22	3881 b ± 649	14	10,784 a ± 2478	14	8713 ab ± 2376	5364	0.015
Time from the beginning of that probe to 1st E2 ^7^	22	1411 b ± 85.9	12	1572 ab ± 168.8	12	2106 a ± 345	556.1	0.026
Before 1st sap ingestion phase E2 > 10 min								
Time to from start of EPG 1st E2 > 10 min ^8^	22	4180 b ± 654	14	10,835 a ± 2488	14	10,709 a ± 2613	5619.6	0.012
Time from 1st probe to 1st E2 > 10 min ^9^	22	4098 b ± 654	14	10,795 a ± 2480	14	10,578 a ± 2568	5564.6	0.011
Time from the beginning of that probe to 1st E2 > 10 min. ^10^	22	1411 b ± 85.9	12	1572 ab ± 168.8	12	2106 a ± 345	556.1	0.026
After 1st phloem phase								
Number of probes after 1st E ^4^	22	9.6 a ± 1.9	12	9.7 a ± 2.9	12	12.5 a ± 2.6	7.52	0.645
Number of probes shorter than 3 min after 1st E ^4^	22	4.8 a ± 1.2	12	5.9 a ± 2.3	12	6 a ± 1.6	5.174	0.84
Potential E2 index ^11^	22	66.9 a ± 4.3	12	44.5 b ± 8.2	12	32.4 b ± 6.1	18.47	<0.001

^1^ Number of replications; ^2^ total duration of EPG recording if E is missing; ^3^ time from 1st probe to the end of EPG recording if E is missing; ^4^ missing data if E is missing; E2; ^5^ total duration of EPG recording if E is missing; ^6^ time from 1st probe to the end of EPG recording if E is missing; ^7^ missing data if E is missing; ^8^ total duration of EPG recording if E is missing; ^9^ time from 1st probe to the end of EPG recording if E is missing; ^10^ missing data if E is missing; ^11^ potential E2 index = the percentage of time spent in E2 by an aphid with any sustained E2, after reaching the first sustained E2. Time and duration of various stylet activities are given in seconds. LSD_0.05_—least significant difference at the 0.05 significance level. Different letters in rows show significant differences at *p* < 0.05 (ANOVA). Shading indicates variables that are significantly different between treatments.

**Table 3:** Probing behavior of *Rhopalosiphum padi* on *Avena sativa* treated with ethanolic solutions of hesperidin: non-sequential EPG parameters.

EPG Variable	Hesperidin 0.0%		Hesperidin 0.1%		Hesperidin 0.5%		LSD_0.05_	*p*-ANOVA
	n ^1^	Mean ± SEM	n ^1^	Mean ± SEM	n ^1^	Mean ± SEM		
No probing								
Total duration of np	15	2232 b ± 385	15	5862 a ± 1389	12	3052 ab ± 1246	3347.69	0.049
Number of np	15	9.5 a ± 6.0	15	11.0 a ± 6.4	12	6.6 a ± 5.0	4.84	0.16
Mean duration of np	15	244.2 a ± 23.8	15	802.9 a ± 293.7	12	651.9 a ± 265.6	694.04	0.186
Probing								
Total probing time	15	26,568 a ± 385	15	22,933 b ± 1388	12	25,747 ab ± 1246	3345.89	0.048
Number of probes	15	9.3 a ± 1.5	15	10.7 a ± 1.7	12	6.3 a ± 1.4	4.82	0.167
Number of short probes (C < 3 min)	15	3.1 a ± 0.8	15	3.1 a ± 0.8	12	1.9 a ± 0.7	2.51	0.526
Pathway phase								
Total duration of C	15	6680 a ± 906.9	15	7707 a ± 798.7	12	5896 a ± 1021.5	2787.51	0.384
Number of C	15	15.4 a ± 2.026	15	17.1 a ± 1.8	12	14.2 a ± 2.6	6.56	0.637
Mean duration of C	15	439.3 a ± 47.75	15	463 a ± 31.8	12	486.3 a ± 52.6	135.85	0.763
Proportion of probing spent in C (%)	15	25.8 a ± 3.8	15	34.1 a ± 3.1	12	23.6 a ± 4	11.13	0.111
Derailed stylet activities								
Total duration of F	15	4115 a ± 840	15	4237 a ± 1191	12	2925 a ± 1109	3260.9	0.651
Number of F	15	1.9 a ± 0.4	15	2 a ± 0.5	12	1.4 a ± 0.5	1.44	0.651
Mean duration of F ^2^	12	2581 a ± 447.5	12	1856 a ± 315.5	7	2477 a ± 836.6	1312.5	0.412
Proportion of probing spent in F (%)	15	15.7 a ± 3.3	15	18.34 a ± 4.72	12	12.4 a ± 5.074	13.45	0.645
Xylem phase								
Total duration of G	15	1401 b ± 815	15	4619 ab ± 837	12	8719 a ± 2470	4370.05	0.004
Number of G	15	0.5 b ± 0.3	15	1.7 ab ± 0.3	12	2.8 a ± 0.8	1.38	0.006
Mean duration of G ^2^	4	2528 a ± 1031	12	3340 a ± 669.3	9	3460 a ± 845	2004.33	0.54
Proportion of probing spent in G (%)	15	5.4 b ± 3.094	15	23.7 a ± 5	12	33.4 a ± 9.2	17.94	0.006
Phloem phase: general								
Total duration of phloem phase E (E1 + E2)	15	14,371 a ± 1904	15	6371 b ± 1979	12	8207 ab ± 2463	6456.76	0.021
Total duration of E1	15	219.9 a ± 49.4	15	175 a ± 67.9	12	395 a ± 209.7	356.87	0.401
Total duration of E2	15	14,151 a ± 1920	15	6196 b ± 1951	12	7812 ab ± 2505	6479.77	0.022
Phloem phase: salivation (E1)								
Number of E1	15	4.3 a ± 0.7	15	3.3 a ± 0.8	12	4.3 a ± 1.1	2.56	0.615
Mean duration of E1 ^2^	15	47.8 a ± 7.64	12	44.2 a ± 8	10	91 a ± 47.5	67.31	0.279
Number of single E1 ^2^	15	0.8 a ± 0.3	15	0.4 a ± 0.2	12	1 a ± 0.5	0.97	0.404
Total duration of E1 followed by E2 ^2^	15	183.2 a ± 43.5	12	136.9 a ± 30.1	10	249.9 a ± 110.3	180.55	0.417
Total duration of E1 followed by E2 > 10 min ^2^	14	99.5 a ± 33.9	9	67.9 a ± 11.6	8	84.1 a ± 22.1	78.49	0.66
Duration of the E1 followed by 1st E2 ^2^	15	41.1 a ± 8.3	12	35.4 a ± 3.3	10	41.5 a ± 8.9	21.63	0.788
Duration of the E1 followed by 1st E2 > 10 min ^2^	14	42.8 a ± 4.5	9	43.2 a ± 4.5	8	37.2 a ± 4.1	12.27	0.521
Contribution of E1 to phloem phase (%) ^2^	15	7.2 a ± 5.1	12	13.6 a ± 5.5	10	14.9 a ± 7.9	17.52	0.587
Proportion of probing spent in E1 (%)	15	0.8 a ± 0.2	15	0.7 a ± 0.2	12	2 a ± 1.3	2.01	0.357
Phloem phase: sap ingestion (E2)								
Number of E2	15	3.5 a ± 0.5	15	2.8 a ± 0.7	12	3.3 a ± 0.9	2.04	0.718
Number of E2 > 10 min	15	1.6 a ± 0.3	15	0.9 a ± 0.3	12	1.3 a ± 0.3	0.88	0.246
Mean duration of E2 ^2^	15	7109 a ± 2091	12	3246 a ± 1566	10	5154 a ± 2277	5881.65	0.34
Duration of the longest E2 ^2^	15	12,299 a ± 2064	12	6619 a ± 2150	10	6895 a ± 2120	6217.1	0.085
Proportion of probing spent in E2 (%)	15	52.2 a ± 6.6	15	23.2 b ± 6.9	12	28.5 b ± 8.7	22.68	0.015

^1^ Number of replications; ^2^ only the EPG recordings that included a particular waveform were included in calculations; np—no probing (aphid stylets outside the plant tissues); C—pathway activity (extracellular stylet penetration with potential drops, i.e., short cell punctures); F—derailed stylet activities (difficulties in penetration); G—xylem phase (ingestion of xylem sap); E—phloem phase including E1 (phloem salivation) and E2 (phloem sap ingestion); E2 > 10 min—sustained ingestion of phloem sap. Time and duration given in seconds. LSD_0.05_—least significant difference at the 0.05 significance level. Different letters in rows show significant differences at *p* < 0.05 (ANOVA). Shading indicates variables that are significantly different between treatments.

**Table 4:** Probing behavior of *Rhopalosiphum padi* on *Avena sativa* treated with ethanolic solutions of hesperidin: sequential EPG parameters.

EPG Variable	Hesperidin 0.0%		Hesperidin 0.1%		Hesperidin 0.5%		LSD_0.05_	*p*-ANOVA
	n ^1^	Mean ± SEM	n ^1^	Mean ± SEM	n ^1^	Mean ± SEM		
Start of EPG								
Time to 1st probe from start of EPG	15	225.4 a ± 75	15	176.9 a ± 85.5	12	458.1 a ± 210.4	386.9	0.276
Duration of 1st probe	15	4874 a ± 1760	15	4416 a ± 1073	12	6467 a ± 2949	5973.42	0.752
Duration of the second nonprobe period	15	239.5 a ± 96.9	15	163.8 a ± 35.3	12	136.5 a ± 52.5	214.22	0.559
Duration of 2nd probe	15	4004 a ± 1722.3	15	2136 a ± 642.2	12	1268 a ± 573.9	3697.24	0.271
Before 1st phloem phase								
Time from start of EPG to 1st E ^2^	15	6812 a ± 1188	15	10,463 a ± 2626	12	10,174 a ± 2846	7013.94	0.442
Time from 1st probe to 1st E ^3^	15	6587 a ± 1180	15	10,286 a ± 2631	12	9716 a ± 2731	6909.23	0.442
Time from the beginning of that probe to 1st E ^4^	15	1907 a ± 717.7	12	2905 a ± 652.7	10	2944 a ± 1134.7	2382.01	0.553
Number of probes to the 1st E1 ^4^	15	3.4 a ± 0.5	12	2.7 a ± 0.9	10	3.6 a ± 0.8	2.05	0.641
Duration of nonprobe period before the 1st E	15	722 a ± 123.9	15	2004 a ± 711.9	12	789 a ± 224.9	1426.15	0.092
Duration of the shortest C wave before E1 ^4^	15	823 b ± 133.5	12	1879 a ± 570.9	10	708 b ± 136.3	990.3	0.024
1st phloem phase								
Duration of 1st phloem phase E ^4^	15	5887 a ± 2382	12	2644 a ± 1623	10	3451 a ± 2221	6337.09	0.492
Before 1st sap ingestion phase E2								
Time from start of EPG to 1st E2 ^5^	15	7051 a ± 1170	15	10,491 a ± 2623	12	11,222 a ± 2926	7074.03	0.393
Time from 1st probe to 1st E2 ^6^	15	6825 a ± 1162	15	10,314 a ± 2628	12	10,764 a ± 2819	6975.26	0.404
Time from the beginning of that probe to 1st E2 ^7^	15	2145 a ± 744.1	12	2940 a ± 651.7	10	1301 a ± 536.4	2003.33	0.229
Before 1st sap ingestion phase E2 > 10 min								
Time to from start of EPG 1st E2 > 10 min ^8^	15	11,488 a ± 2051	15	18,381 a ± 2635	12	16,746 a ± 2979	7836.54	0.132
Time from 1st probe to 1st E2 > 10 min ^9^	15	11,262 a ± 2065	15	18,204 a ± 2657	12	16,288 a ± 2909	7817.67	0.132
Time from the beginning of that probe to 1st E2 > 10 min. ^10^	14	1301 b ± 203.2	9	2972 ab ± 760.1	8	3358 a ± 1075.7	1686.75	0.027
After 1st phloem phase								
Number of probes after 1st E	15	5.9 a ± 1.3	15	4.5 a ± 1.1	12	2.8 a ± 1.2	3.74	0.236
Number of probes shorter than 3 min after 1st E	15	2.3 a ± 0.7	15	1 ab ± 0.4	12	0.7 b ± 0.4	1.57	0.046
Potential E2 index ^11^	15	63 a ± 8.0	9	26.0 b ± 8.4	8	39.3 ab ± 11.9	28.62	0.019

^1^ Number of replications; ^2^ total duration of EPG recording if E is missing; ^3^ time from 1st probe to the end of EPG recording if E is missing; ^4^ missing data if E is missing; E2; ^5^ total duration of EPG recording if E is missing; ^6^ time from 1st probe to the end of EPG recording if E is missing; ^7^ missing data if E is missing; ^8^ total duration of EPG recording if E is missing; ^9^ time from 1st probe to the end of EPG recording if E is missing; ^10^ missing data if E is missing; ^11^ Potential E2 index = the percentage of time spent in E2 by an aphid with any sustained E2, after reaching the first sustained E2. Time and duration of various stylet activities are given in seconds. LSD_0.05_—least significant difference at the 0.05 significance level. Different letters in rows show significant differences at *p* < 0.05 (ANOVA). Shading indicates variables that are significantly different between treatments.

**Table 5:** Probing behavior of *Myzus persicae* on *Brassica rapa* subsp. *pekinensis* treated with ethanolic solutions of hesperidin: non-sequential EPG parameters.

EPG Variable	Hesperidin 0.0%		Hesperidin 0.1%		Hesperidin 0.5%		LSD_0.05_	*p*-ANOVA
	n ^1^	Mean ± SEM	n ^1^	Mean ± SEM	n ^1^	Mean ± SEM		
No probing								
Total duration of np	17	3614 a ± 777.3	15	5218 a ± 1186.3	14	4424 a ± 901.9	2866	0.489
Number of np	17	41.2 a ± 6.6	15	37 a ± 5.1	14	33.4 a ± 5.8	17.78	0.655
Mean duration of np	17	82.2 b ± 6.6	15	146.3 a ± 33.2	14	129.2 ab ± 16.2	62.7	0.044
Probing								
Total probing time	17	25,186 a ± 777.3	15	23,580 a ± 1186.9	14	24,375 a ± 901.9	2866.7	0.489
Number of probes	17	41.1 a ± 6.6	15	36.7 a ± 5.1	14	33.3 a ± 5.7	17.71	0.65
Number of short probes (C < 3 min)	17	26.9 a ± 5.2	15	23.2 a ± 4.1	14	20.6 a ± 4.6	14.02	0.633
Pathway phase								
Total duration of C	17	11,757 a ± 1358	15	13,334 a ± 1399	14	12,617 a ± 1924	4629.2	0.764
Number of C	17	43.7 a ± 6.7	15	39.8 a ± 5.2	14	35.8 a ± 5.9	18.14	0.656
Mean duration of C	17	344.7 a ± 50.9	15	394.3 a ± 50.8	14	461.1 a ± 97.2	200.2	0.477
Proportion of probing spent in C (%)	17	49 a ± 6.6	15	58.3 a ± 5.9	14	53.9 a ± 8.3	20.8	0.635
Derailed stylet activities								
Total duration of F	17	1750.8 a ± 585.2	15	535.6 ab ± 359.8	14	92.5 b ± 64.9	1279.3	0.023
Number of F	17	0.7 a ± 0.2	15	0.3 ab ± 0.2	14	0.1 b ± 0.1	0.516	0.031
Mean duration of F ^2^	9	2526 a ± 552.4	3	2274 a ± 1389.2	2	648 b ± 155.5	1453.9	0.03
Proportion of probing spent in F (%)	17	6.8 a ± 2.4	15	2 ab ± 1.345	14	0.4 b ± 0.3	5.142	0.027
Xylem phase								
Total duration of G	17	764 b ± 236.3	15	2321 a ± 505.8	14	1198 ab ± 601.7	1349.7	0.05
Number of G	17	0.6 a ± 0.2	15	1 a ± 0.2	14	0.5 a ± 0.2	0.586	0.205
Mean duration of G ^2^	8	1235 b ± 115.6	11	2404 a ± 372.3	6	2103 a ± 505	812.6	0.011
Proportion of probing spent in G (%)	17	3.1 b ± 0.9	15	9.6 a ± 2.3	14	5.4 ab ± 3	6.4	0.049
Phloem phase: general								
Total duration of phloem phase E (E1 + E2)	17	10,914 a ± 1842	15	7390 a ± 1645	14	10,467 a ± 2513	5972.4	0.406
Total duration of E1	17	100.1 a ± 18.83	15	174 a ± 61.5	14	114.6 a ± 21.2	114.6	0.359
Total duration of E2	17	10,814 a ± 1850	15	7216 a ± 1659	14	10,353 a ± 2516	5996.7	0.394
Phloem phase: salivation (E1)								
Number of E1	17	1.9 a ± 0.3	15	2.5 a ± 0.4	14	2.4 a ± 0.5	1.186	0.519
Mean duration of E1 ^2^	16	51 a ± 4.2	14	60.6 a ± 7.6	13	48.6 a ± 4.1	15.99	0.265
Number of single E1	17	0.1 a ± 0.1	15	0.1 a ± 0.1	14	0.1 a ± 0.1	0.2654	0.979
Total duration of E1 followed by E2 ^2^	16	99.8 a ± 18.2	14	180.4 a ± 65	13	116.2 a ± 19.9	115.3	0.302
Total duration of E1 followed by E2 > 10 min ^2^	15	62.8 b ± 7.9	12	106.6 a ± 11	13	79.4 ab ± 11.8	28.31	0.007
Duration of the E1 followed by 1st E2 ^2^	16	57.4 a ± 7.3	14	56.5 a ± 5.2	13	48.8 a ± 3.3	16.83	0.515
Duration of the E1 followed by 1st E2 > 10 min ^2^	15	52.2 ab ± 3.5	12	61.2 a ± 5.1	13	48.4 b ± 3.2	11.02	0.05
Contribution of E1 to phloem phase (%) ^2^	16	3.3 a ± 1.8	14	6.1 a ± 2.7	13	3.1 a ± 0.9	5.71	0.468
Proportion of probing spent in E1 (%)	17	0.4 a ± 0.1	15	0.8 a ± 0.3	14	0.5 a ± 0.1	0.532	0.292
Phloem phase: sap ingestion (E2)								
Number of E2	17	1.8 a ± 0.3	15	2.4 a ± 0.4	14	2.3 a ± 0.5	1.156	0.525
Number of E2 > 10 min	17	1.1 a ± 0.2	15	1.5 a ± 0.3	14	1.6 a ± 0.3	0.687	0.297
Mean duration of E2 ^2^	16	9315 a ± 2105	14	4221 a ± 1268	13	7348 a ± 2636	5966.5	0.194
Duration of the longest E2 ^2^	16	10,798 a ± 1885	14	6126 a ± 1615	13	9363 a ± 2480	5776.2	0.221
Proportion of probing spent in E2 (%)	17	40.6 a ± 6.7	15	29.3 a ± 5.9	14	39.7 a ± 8.9	21.47	0.475

^1^ Number of replications; ^2^ only the EPG recordings that included a particular waveform were included in calculations; np—no probing (aphid stylets outside the plant tissues); C—pathway activity (extracellular stylet penetration with potential drops, i.e., short cell punctures); F—derailed stylet activities (difficulties in penetration); G—xylem phase (ingestion of xylem sap); E—phloem phase including E1 (phloem salivation) and E2 (phloem sap ingestion); E2 > 10 min—sustained ingestion of phloem sap. Time and duration given in seconds. LSD_0.05_—least significant difference at the 0.05 significance level. Different letters in rows show significant differences at *p* < 0.05 (ANOVA). Shading indicates variables that are significantly different between treatments.

**Table 6:** Probing behavior of *Myzus persicae* on *Brassica rapa* subsp. *pekinensis* treated with ethanolic solutions of hesperidin: sequential EPG parameters.

EPG Variable	Hesperidin 0.0%		Hesperidin 0.1%		Hesperidin 0.5%		LSD_0.05_	*p*-ANOVA
	n ^1^	Mean ± SEM	n ^1^	Mean ± SEM	n ^1^	Mean ± SEM		
Start of EPG								
Time to 1st probe from start of EPG	17	74.6 a ± 28.9	15	107.19 a ± 64.3	14	91.4 a ± 31.2	131.1	0.867
Duration of 1st probe	17	138.5 a ± 44	15	76 a ± 22.6	14	80 a ± 31	104.5	0.359
Duration of the second nonprobe period	17	88.8 a ± 21.2	15	59.2 a ± 14.1	14	90.3 a ± 14.7	52.5	0.383
Duration of 2nd probe	17	1356 a ± 1013.7	15	163.4 a ± 69.2	14	271.1 a ± 144.6	1960.1	0.355
Before 1st phloem phase								
Time from start of EPG to 1st E ^2^	17	11,063 a ± 1829	15	11,764 a ± 2326	14	10,917 a ± 2413	6478.2	0.959
Time from 1st probe to 1st E ^3^	17	10,989 a ± 1831	15	11,657 a ± 2332	14	10,825 a ± 2414	6488	0.961
Time from the beginning of that probe to 1st E ^4^	16	2156 a ± 538.5	14	2435 a ± 465.5	13	1939 a ± 398	1396.8	0.765
Number of probes to the 1st E1	16	20.7 a ± 3.4	14	18.4 a ± 4.5	13	20.2 a ± 4.8	12.09	0.916
Duration of nonprobe period before the 1st E	17	2274 a ± 756.6	15	1952 a ± 524.9	14	2356 a ± 631.3	1966.2	0.903
Duration of the shortest C wave before E1	16	1987 a ± 555.5	14	1994 a ± 399.5	13	1587 a ± 264.8	1289.9	0.759
1st phloem phase								
Duration of 1st phloem phase E	16	8754 a ± 2254	14	2444 b ± 1230	13	7831 ab ± 2644	6140.1	0.045
Before 1st sap ingestion phase E2								
Time from start of EPG to 1st E2 ^5^	17	11,497 a ± 1828	15	12,694 a ± 2220	14	10,962 a ± 2412	6369.9	0.848
Time from 1st probe to 1st E2 ^6^	17	11,422 a ± 1832	15	12,587 a ± 2229	14	10,871 a ± 2413	6383.3	0.852
Time from the beginning of that probe to 1st E2 ^7^	16	2256 a ± 531.8	14	2571 a ± 453.3	13	1988 a ± 397.9	1376.9	0.686
Before 1st sap ingestion phase E2 > 10 min								
Time to from start of EPG 1st E2 > 10 min ^8^	17	15,197 a ± 2152	15	16,349 a ± 2601	14	11,384 a ± 2543	7219.9	0.346
Time from 1st probe to 1st E2 > 10 min ^9^	17	15,123 a ± 2150	15	16,242 a ± 2616	14	11,293 a ± 2544	7233.6	0.348
Time from the beginning of that probe to 1st E2 > 10 min. ^10^	15	2495 a ± 546.4	12	3056 a ± 552.8	13	2027 a ± 389.9	1417.5	0.335
After 1st phloem phase								
Number of probes after 1st E ^10^	17	15.7 a ± 4.6	15	15.7 a ± 4.3	14	11.6 a ± 3.2	12.48	0.733
Number of probes shorter than 3 min after 1st E ^10^	17	10.7 a ± 3.6	15	10.3 a ± 3.3	14	7.8 a ± 2.8	9.87	0.802
Potential E2 index ^11^	17	60.9 a ± 9.2	12	48.8 a ± 9.8	13	51.3 a ± 9.7	28.66	0.629

^1^ Number of replications; ^2^ total duration of EPG recording if E is missing; ^3^ time from 1st probe to the end of EPG recording if E is missing; ^4^ missing data if E is missing; E2; ^5^ total duration of EPG recording if E is missing; ^6^ time from 1st probe to the end of EPG recording if E is missing; ^7^ missing data if E is missing; ^8^ total duration of EPG recording if E is missing; ^9^ time from 1st probe to the end of EPG recording if E is missing; ^10^ missing data if E is missing; ^11^ potential E2 index = the percentage of time spent in E2 by an aphid with any sustained E2, after reaching the first sustained E2. Time and duration of various stylet activities are given in seconds. LSD_0.05_—least significant difference at the 0.05 significance level. Different letters in rows show significant differences at *p* < 0.05 (ANOVA). Shading indicates variables that are significantly different between treatments.

## Data Availability

All original data are available from authors upon request.

## Supplementary Materials

The following supporting information can be downloaded at: https://www.mdpi.com/article/10.3390/ijms25094822/s1.
