# Supplementary material for: Hesperidin as a Species-Specific Modifier of Aphid Behavior
Source: Int J Mol Sci. 2024 Apr 28;25(9):4822. doi: 10.3390/ijms25094822 (PMC11084187; doi:10.3390/ijms25094822)
Supplement: Supplementary file 1 [file ijms-25-04822-s001.zip › ijms-2973028-supplementary.pdf]

Table S1. Probing behavior of *Acyrthosiphon pisum* on *Pisum sativum* treated with ethanolic solutions of hesperidin: non-sequential EPG parameters (min-max and median values).

EPG variable	Hesperidin 0.0 %			Hesperidin 0.1 %			Hesperidin 0.5 %		
	n[1]	Min-Max	Median	n[1]	Min-Max	Median	n[1]	Min-Max	Median
**No probing**									
Total duration of np	22	130.1-3619	1012	14	105.5-10686	2078	14	204.6-19108	1981
Number of np	22	2-38	14.5	14	5-57	15.5	14	7-46	15.5
Mean duration of np	22	37.52-185	74.4	14	15.07-1069	97.05	14	29.23-1911	114.3
**Probing**									
Total probing time	22	25181-28670	27788	14	18114-28695	26722	14	9692-28595	26819
Number of probes	22	2-38	14.5	14	5-57	15.5	14	7-46	15.5
Number of short probes (C<3 minutes)	22	0-25	7	14	2-39	7	14	1-31	6.5
**Pathway phase**									
Total duration of C	22	2271-19449	10666	14	4176-27334	13853	14	3545-24719	13573
Number of C	22	4-44	16.5	14	6-59	19	14	9-48	20
Mean duration of C	22	141.9-978	594.2	14	208.8-2485	683.6	14	348.4-1274	639
**Derailed stylet activities**									
Total duration of F	22	0-0	0	14	0-0	0	14	0-0	0
Number of F	22	0-0	0	14	0-0	0	14	0-0	0
Mean duration of F[2]	22	0-0	0	14	0-0	0	14	0-0	0
**Xylem phase**									
Total duration of G	22	0-0	0	14	0-12300	0	14	0-23655	0
Number of G	22	0-0	0	14	0-2	0	14	0-5	0
Mean duration of G[2]	22	0-0	0	6	1040-6150	3763	2	3526-11827	7677
**Phloem phase: general**									
Total duration of phloem phase E (E1+E2)	22	7287-25629	16982	14	0-22925	9249	14	0-16754	7235
Total duration of E1	22	42.79-1924	291.7	14	0-1724	258.7	14	0-2395	485.1
Total duration of E2	22	6567-25586	15878	14	0-22624	8920	14	0-16641	5909
**Phloem phase: salivation (E1)**									
Number of E1	22	1-27	7.5	14	0-28	4.5	14	0-14	6
Mean duration of E1[2]	22	26.894-245.3	55.58	12	0-246.2	47.9	12	0-180.9	74.29
Number of single E1[2]	22	0-2	0	12	0-1	0	12	0-1	0
Total duration of E1 followed by E2[2]	22	42.79-1129.2	277.7	12	116.8-748	265.8	12	62.72-1069.5	358.6
Total duration of E1 followed by E2 >10 min[2]	22	42.79-519.2	171.4	12	58.6-637.8	216.1	12	62.72-572.6	175.2
Duration of the E1 followed by 1st E2[2]	22	23.12-418.8	41.42	12	37.85-394.4	73.45	12	36.92-280.9	66.18
Duration of the E1 followed by 1st E2 >10 min[2]	22	23.12-418.8	42.69	12	37.85-394.4	70.66	12	36.92-303.7	85.75
Contribution of E1 to phloem phase (%)[2]	22	0.167-11.15	2.843	14	0.7121-19.19	4.589	14	0.6694-30.55	5.007
**Phloem phase: sap ingestion (E2)**									
Number of E2	22	1-21	6.5	14	0-26	4	14	0-8	4.5
Number of E2 >10 min	22	1-8	3	14	0-7	3.5	14	0-8	2
Mean duration of E2[2]	22	730.2-25586	1916	12	0-5656	1296	12	0-8321	1290
Duration of the longest E2[2]	22	1374-25586	7108	12	1383-10131	3548	12	1885-11158	3536

[1] number of replications; [2] only the EPG recordings that included a particular waveform were included in calculations; np – no probing (aphid stylets outside the plant tissues); C – pathway activity (extracellular stylet penetration with potential drops, i.e., short cell punctures); F – derailed stylet activities (difficulties in penetration); G – xylem phase (ingestion of xylaem sap); E – phloem phase including E1 (phloem salivation) and E2 (phloem sap ingestion); E2 > 10 min. – sustained ingestion of phloem sap.

Table S2. Probing behavior of *Acyrthosiphon pisum* on *Pisum sativum* treated with ethanolic solutions of hesperidin: sequential EPG parameters (min-max and median values).

EPG variables	Hesperidin 0.0 %			Hesperidin 0.1 %			Hesperidin 0.5 %		
	n[1]	Min-Max	Median	n[1]	Min-Max	Median	n[1]	Min-Max	Median
**Start of EPG**									
Time to 1st probe from start of EPG	22	0.39-448.1	48.9	14	2.59-181	19.73	14	0.53-1258.6	51.73
Duration of 1st probe	22	3.87-13377	112.09	14	3.39-1591	17.61	14	11.08-6213	75.05
Duration of the second nonprobe period	22	17.43-196.3	42.28	14	10.48-1455.6	35.08	14	9.69-206.3	51.67
Duration of 2nd probe	22	9.56-15293	539.7	14	2.89-7238	24.4	14	7.6-20044	104.2
**Before 1st phloem phase**									
Time from start of EPG to 1st E[2]	22	676-11760	2786	14	1865-28800	7190	14	782-28800	5079
Time from 1st probe to 1st E[3]	22	622-11604	2776	14	1859-28770	7172	14	735-28799	5034
Time from the beginning of that probe to 1st E[4]	22	578.7-1887	1334	12	343.9-2454	1341	12	533.4-4536	1270
Number of probes to the 1st E1	22	1-16	4.5	12	2-23	8.5	12	1-30	4
Duration of nonprobe period before the 1st E	22	0.58-2673	192	14	51.62-10686	1163.1	14	11.17-3813	473.4
Duration of the shortest C wave before E1	22	453.5-1555	1021	12	343.9-2043	1283	12	533.4-2021	1268
**1st phloem phase**									
Duration of 1st phloem phase E	22	211.5-25629	2838	12	162.8-9792	1778	12	49.4-3472	1423
**Before 1st sap ingestion phase E2**									
Time from start of EPG to 1st E2[5]	22	1450-12178	2839	14	1925-28800	7246	14	1038-28800	5396
Time from 1st probe to 1st E2[6]	22	668-12023	2819	14	1919-28770	7228	14	1016-28799	5114
Time from the beginning of that probe to 1st E2[7]	22	669-2419	1396	12	411.4-2492	1491	12	667.5-4573	1817
**Before 1st sap ingestion phase E2>10 min**									
Time to from start of EPG 1st E2> 10 min[8]	22	1450-12178	2839	14	1925-28800	7246	14	1038-28800	5396
Time from 1st probe to 1st E2>10 min[9]	22	1208-12023	2819	14	1919-28770	7228	14	1016-28799	5324
Time from the beginning of that probe to 1st E2> 10 min.[10]	22	669-2419	1396	12	411.4-2492	1491	12	667.5-4573	1817
**After 1st phloem phase**									
Number of probes after 1st E	22	0-33	7	12	0-35	6.5	12	0-36	10
Number of probes shorter than 3 minutes after 1st E	22	0-21	3	12	0-26	2.5	12	0-18	3.5
Potential E2 index	22	24.916-100	62.06	12	0-99.86	49.69	12	0-65.25	29.43

[1] number of replications; [2] total duration of of EPG recording if E is missing; [3] time from 1st probe to the end of EPG recording if E is missing; [4] missing data if E is missing; E2; [5] total duration of of EPG recording if E is missing; [6] time from 1st probe to the end of EPG recording if E is missing; [7] missing data if E is missing; [8] total duration of of EPG recording if E is missing; [9] time from 1st probe to the end of EPG recording if E is missing; [10] missing data if E is missing.

Table S3. Probing behavior of *Rhopalosiphum padi* on *Avena sativa* treated with ethanolic solutions of hesperidin: non-sequential EPG parameters (min-max and median values).

EPG variable		Hesperidin 0.0 %			Hesperidin 0.1 %			Hesperidin 0.5 %	
	n[1]	Min-Max	Median	n[1]	Min-Max	Median	n[1]	Min-Max	Median
**No probing**									
Total duration of np	15	270.7-5108	2207	15	674.3-17360	4985	12	406-12287	1392
Number of np									
Mean duration of np	15	30.08-378	246.9	15	74.46-3472	308.5	12	78.27-2694	244.6
**Probing**									
Total probing time	15	23691-28529	26593	15	11440-28125	23815	12	16513-28394	27408
Number of probes	15	3-19	7	15	4-22	10	12	1-17	5
Number of short probes (C<3 minutes)	15	0-9	2	15	0-9	2	12	0-8	1
**Pathway phase**									
Total duration of C	15	1101-12904	7172	15	3009-12759	8103	12	1532-13887	5568
Number of C	15	4-32	12	15	7-29	18	12	2-32	14
Mean duration of C	15	213.8-806.5	404.7	15	289.4-750.5	448.4	12	229.7-921.8	492.7
**Derailed stylet activities**									
Total duration of F	15	0-11941	3738	15	0-15032	2217	12	0-10652	716
Number of F	15	0-5	2	15	0-7	2	12	0-5	1
Mean duration of F[2]	12	617.4-5970	2180	12	0-3487	2174	7	602.8-5641	1346
**Xylem phase**									
Total duration of G	15	0-11154	0	15	0-10154	4649	12	0-24573	5557
Number of G	15	0-3	0	15	0-4	2	12	0-8	2.5
Mean duration of G[2]	4	1195-5577	1670	12	892-10049	2701	9	1315-8191	2467
**Phloem phase: general**									
Total duration of phloem phase E (E1+E2)	15	173.63-25309	16131	15	0-19658	2025	12	0-26550	6008
Total duration of E1	15	26.08-591	175.1	15	0-1022	79.3	12	0-2574	152.6
Total duration of E2	15	37.12-25263	16102	15	0-19609	1722	12	0-26497	5682
**Phloem phase: salivation (E1)**									
Number of E1	15	1-10	4	15	0-8	2	12	0-11	3.5
Mean duration of E1[2]	15	22.8-136	37.63	12	23.66-127.7	37.93	10	23.4-514.8	40.2
Number of single E1[2]	15	0-3	0	15	0-2	0	12	0-6	0.5
Total duration of E1 followed by E2[2]	15	26.08-544	175.1	12	23.69-368.8	133.4	10	30.29-1207.3	152.8
Total duration of E1 followed by E2 >10 min[2]	14	24.52-513.6	58.31	9	28.88-128.7	56.9	8	30.29-212.9	54.57
Duration of the E1 followed by 1st E2[2]	15	20.08-151.4	33.39	12	23.69-56	30.31	10	21.83-116.9	31.79
Duration of the E1 followed by 1st E2 >10 min[2]	14	24.52-88.07	37.89	9	28.88-68.94	42.58	8	26.81-56.21	31.44
Contribution of E1 to phloem phase (%)[2]	15	0.106-78.62	1.24	12	0.246-58.39	5.141	10	0.1478-72.5	4.167
**Phloem phase: sap ingestion (E2)**									
Number of E2	15	1-7	3	15	0-7	2	12	0-10	2.5
Number of E2 >10 min	15	0-4	1	15	0-3	1	12	0-3	1
Mean duration of E2[2]	15	37.12-24570	3512	12	16.87-19609	1996	10	91.44-20461	1436
Duration of the longest E2[2]	15	37.12-25225	11388	12	23.9-19609	3383	10	149.01-20461	5990

[1] number of replications; [2] only the EPG recordings that included a particular waveform were included in calculations; np – no probing (aphid stylets outside the plant tissues); C – pathway activity (extracellular stylet penetration with potential drops, i.e., short cell punctures); F – derailed stylet activities (difficulties in penetration); G – xylem phase (ingestion of xylem sap); E – phloem phase including E1 (phloem salivation) and E2 (phloem sap ingestion); E2 > 10 min. – sustained ingestion of phloem sap.

Table S4. Probing behavior of *Rhopalosiphum padi* on *Avena sativa* treated with ethanolic solutions of hesperidin: sequential EPG parameters (min-max and median values).

EPG variable	Hesperidin 0.0 %			Hesperidin 0.1 %			Hesperidin 0.5 %		
	n[1]	Min-Max	Median	n[1]	Min-Max	Median	n[1]	Min-Max	Median
**Start of EPG**									
Time to 1st probe from start of EPG	15	0.96-1166	130.2	15	0.16-1319	47.1	12	15.98-2694	213.6
Duration of 1st probe	15	9.63-26288	3768	15	10.19-14289	4441	12	42.7-28394	676
Duration of the second nonprobe period	15	13.72-1505.7	131.91	15	5.16-490.1	115.25	12	9.63-466.9	42.7
Duration of 2nd probe	15	11.26-19773	493.8	15	14.74-7398	553.8	12	16.63-6083	579.5
**Before 1st phloem phase**									
Time from start of EPG to 1st E[2]	15	957.2-14900	5886	15	853.9-28800	6747	12	731.3-28800	8043
Time from 1st probe to 1st E[3]	15	956.2-14884	5544	15	711.2-28800	6709	12	626.8-28554	7667
Time from the beginning of that probe to 1st E[4]	15	424.8-11528	956	12	501.5-7360	2931	10	448.1-11400	1280
Number of probes to the 1st E1	15	1-8	3	12	1-11	1	10	1-9	3
Duration of nonprobe period before the 1st E	15	0.96-1538	660.5	15	0.83-8966	549.3	12	104.53-2694	542.3
Duration of the shortest C wave before E1	15	397.6-2525	729.1	12	468.9-7360	1218.4	10	288.5-1515	656.1
**1st phloem phase**									
Duration of 1st phloem phase E	15	14.33-25250	222.3	12	40.29-19658	168.2	10	37.12-20491	82.1
**Before 1st sap ingestion phase E2**									
Time from start of EPG to 1st E2[5]	15	981.7-14938	5932	15	883.4-28800	6773	12	764.6-28800	9241
Time from 1st probe to 1st E2[6]	15	980.7-14922	5590	15	740.8-28800	6736	12	660.1-28554	8865
Time from the beginning of that probe to 1st E2[7]	15	453.5-11992	1023	12	525.2-7389	2959	10	357.1-5995	701
**Before 1st sap ingestion phase E2>10 min**									
Time to from start of EPG 1st E2> 10 min[8]	15	982-28800	10041	15	883-28800	17618	12	1948-28800	16273
Time from 1st probe to 1st E2>10 min[9]	15	981-28784	9760	15	741-28800	17607	12	1542-28619	15951
Time from the beginning of that probe to 1st E2> 10 min.[10]	14	453.5-2760	974	9	740.8-7389	3385	8	357.1-8892	2348
**After 1st phloem phase**									
Number of probes after 1st E	15	0-14	5	15	0-12	3	12	0-13	1.5
Number of probes shorter than 3 minutes after 1st E	15	0-6	1	15	0-4	0	12	0-4	0
Potential E2 index	15	0.26779-100	62.14	9	0-100	7.82	8	0-100	22.28

[1] number of replications; [2] total duration of of EPG recording if E is missing; [3] time from 1st probe to the end of EPG recording if E is missing; [4] missing data if E is missing; E2; [5] total duration of of EPG recording if E is missing; [6] time from 1st probe to the end of EPG recording if E is missing; [7] missing data if E is missing; [8] total duration of of EPG recording if E is missing; [9] time from 1st probe to the end of EPG recording if E is missing; [10] missing data if E is missing.

Table S5. Probing behavior of *Myzus persicae* on *Brassica rapa* subsp. *pekinensis* treated with ethanolic solutions of hesperidin: non-sequential EPG parameters (min-max and median values).

EPG variable	Hesperidin 0.0 %			Hesperidin 0.1 %			Hesperidin 0.5 %		
	n[1]	Min-Max	Median	n[1]	Min-Max	Median	n[1]	Min-Max	Median
**No probing**									
Total duration of np	17	670.8-13314	2359	15	461.1-16363	3548	14	167.7-10158	3910
Number of np	17	12-100	27	15	8-68	35	14	4-65	31.5
Mean duration of np	17	44.54-133.1	81.72	15	49.69-467.5	87.81	14	41.93-267.7	106.63
**Probing**									
Total probing time	17	15486-28129	26441	15	12437-28339	25252	14	18641-28632	24890
Number of probes	17	12-100	27	15	8-68	35	14	4-65	31.5
Number of short probes (C<3 minutes)	17	3-74	14	15	0-50	21	14	1-51	12
**Pathway phase**									
Total duration of C	17	3144-21443	10945	15	5694-22804	13407	14	1186-23659	10923
Number of C	17	13-100	33	15	10-71	39	14	4-70	33
Mean duration of C	17	80.6-809	288.4	15	176.5-862	349.2	14	153.3-1508	355.2
**Derailed stylet activities**									
Total duration of F	17	0-8719	1123.3	15	0-5029	0	14	0-803	0
Number of F	17	0-3	1	15	0-2	0	14	0-1	0
Mean duration of F[2]	9	818.6-5649	2380	3	583.6-5029	1210	2	492.1-803	648
**Xylem phase**									
Total duration of G	17	0-3307	0	15	0-5832	1951.7	14	0-8310	0
Number of G	17	0-3	0	15	0-2	1	14	0-2	0
Mean duration of G[2]	8	902.5-1774	1083	11	1121.1-5094	1967	6	796.5-4155	1946
**Phloem phase: general**									
Total duration of phloem phase E (E1+E2)	17	0-22551	11602	15	0-19408	4846	14	0-26932	7793
Total duration of E1	17	0-302.8	68.2	15	0-1010.3	123.2	14	0-229.1	110.9
Total duration of E2	17	0-22510	11417	15	0-19327	4723	14	0-26908	7627
**Phloem phase: salivation (E1)**									
Number of E1	17	0-4	1	15	0-7	2	14	0-6	2.5
Mean duration of E1[2]	16	35.05-100.9	45.83	14	31.75-144.3	54.4	13	21.45-76.4	46.56
Number of single E1[2]	17	0-1	0	15	0-1	0	14	0-1	0
Total duration of E1 followed by E2[2]	16	35.05-302.8	74.04	14	49.98-1010.3	120.64	13	23.89-229.1	104.95
Total duration of E1 followed by E2 >10 min[2]	15	35.05-147.2	54.41	12	49.98-188.7	96.01	13	23.89-163.5	62.05
Duration of the E1 followed by 1st E2[2]	16	25.48-150	46.62	14	24.04-90.1	53.06	13	23.89-66.9	45.87
Duration of the E1 followed by 1st E2 >10 min[2]	15	35.05-79.13	45.39	12	41.11-90.09	56.37	13	23.89-64.6	45.87
Contribution of E1 to phloem phase (%)[2]	16	0.168-29.36	1.03	14	0.2802-36.81	2.086	13	0.0887-10.93	1.913
**Phloem phase: sap ingestion (E2)**									
Number of E2	17	0-4	1	15	0-7	2	14	0-6	2
Number of E2 >10 min	17	0-3	1	15	0-3	2	14	0-4	1
Mean duration of E2[2]	16	242.9-22510	4272	14	187.1-17789	2524	13	419.4-26908	3281
Duration of the longest E2[2]	16	513.5-22510	10191	14	351-18621	3938	13	601.2-26908	7622

[1] number of replications; [2] only the EPG recordings that included a particular waveform were included in calculations; np – no probing (aphid stylets outside the plant tissues); C – pathway activity (extracellular stylet penetration with potential drops, i.e., short cell punctures); F – derailed stylet activities (difficulties in penetration); G – xylem phase (ingestion of xylaem sap); E – phloem phase including E1 (phloem salivation) and E2 (phloem sap ingestion); E2 > 10 min. – sustained ingestion of phloem sap.

Table S6. Probing behavior of *Myzus persicae* on *Brassica rapa* subsp. *pekinensis* treated with ethanolic solutions of hesperidin: sequential EPG parameters (min-max and median values).

EPG variable	Hesperidin 0.0 %			Hesperidin 0.1 %			Hesperidin 0.5 %		
	n[1]	Min-Max	Median	n[1]	Min-Max	Median	n[1]	Min-Max	Median
**Start of EPG**									
Time to 1st probe from start of EPG	17	0.68-438.4	32.16	15	0.9-995.4	47.73	14	1.04-396.7	52.21
Duration of 1st probe	17	6.34-551.8	46.69	15	0.1-281.4	54.27	14	10.06-393.3	38.49
Duration of the second nonprobe period	17	18.39-309.4	41.28	15	24.72-242.7	46.33	14	25.97-203.1	70.25
Duration of 2nd probe	17	11.5-17472	175.55	15	0-868	30.99	14	9.57-2071	79.1
**Before 1st phloem phase**									
Time from start of EPG to 1st E[2]	17	1473-28800	9438	15	1584-28800	8271	14	1659-28800	8547
Time from 1st probe to 1st E[3]	17	1473-28793	9408	15	1584-28736	8219	14	1635-28787	8349
Time from the beginning of that probe to 1st E[4]	16	425.8-8958	1586	14	416.5-5472	1781	13	715.6-5659	1478
Number of probes to the 1st E1	16	2-50	16.5	14	0-68	16.5	13	3-56	14
Duration of nonprobe period before the 1st E	17	335.5-13314	1530	15	111.1-6352	1106	14	167.7-8872	1847
Duration of the shortest C wave before E1	16	341.5-8958	1132	14	412.4-5127	1306	13	600-3593	1188
**1st phloem phase**									
Duration of 1st phloem phase E	16	56.89-22551	4162	14	31.68-17839	862	13	136.43-26932	2632
**Before 1st sap ingestion phase E2**									
Time from start of EPG to 1st E2[5]	17	1508-28800	10558	15	1628-28800	11011	14	1701-28800	8601
Time from 1st probe to 1st E2[6]	17	1508-28793	10526	15	1627-28736	11010	14	1677-28787	8403
Time from the beginning of that probe to 1st E2[7]	16	451.3-9002	1681	14	495-5513	1984	13	782.6-5705	1536
**Before 1st sap ingestion phase E2>10 min**									
Time to from start of EPG 1st E2> 10 min[8]	17	1508-28800	14644	15	1628-28800	16260	14	1701-28800	8601
Time from 1st probe to 1st E2>10 min[9]	17	1508-28793	14610	15	1627-28798	16212	14	1677-28787	8403
Time from the beginning of that probe to 1st E2> 10 min.[10]	15	773-9002	1762	12	785.7-6252	2888	13	815-5705	1536
**After 1st phloem phase**									
Number of probes after 1st E	17	0-56	12	15	0-57	10	14	0-42	9.5
Number of probes shorter than 3 minutes after 1st E	17	0-48	4	15	0-42	5	14	0-37	4.5
Potential E2 index	17	0-100	55.67	12	0-100	37.88	13	0-100	44.65

[1] number of replications; [2] total duration of of EPG recording if E is missing; [3] time from 1st probe to the end of EPG recording if E is missing; [4] missing data if E is missing; E2; [5] total duration of of EPG recording if E is missing; [6] time from 1st probe to the end of EPG recording if E is missing; [7] missing data if E is missing; [8] total duration of of EPG recording if E is missing; [9] time from 1st probe to the end of EPG recording if E is missing; [10] missing data if E is missing.
